# Supplementary material for: Alpha-Deoxyguanosine to Reshape the Alpha-Thrombin Binding Aptamer
Source: Int J Mol Sci. 2023 May 7;24(9):8406. doi: 10.3390/ijms24098406 (PMC10179326; doi:10.3390/ijms24098406)
Supplement: Supplementary file 1 [file ijms-24-08406-s001.zip › ijms-2339052-supplementary.pdf]

Supplementary information

Alpha-Deoxyguanosine to Reshape the Alpha-Thrombin Binding Aptamer

Natalia A. Kolganova [1,†], Vladimir B. Tsvetkov [2,3,4,†], Andrey A. Stomakhin [1], Sergei A. Surzhikov [1], Edward N. Timofeev [1,*] and Irina V. Varizhuk [1]

[1] Engelhardt Institute of Molecular Biology, Russian Academy of Sciences, 119991 Moscow, Russia

[2] Federal Research and Clinical Center of Physical-Chemical Medicine, 119435 Moscow, Russia

[3] Institute of Biodesign and Complex System Modeling, Sechenov First Moscow State Medical University, 119146 Moscow, Russia

[4] A.V. Topchiev Institute of Petrochemical Synthesis, Russian Academy of Sciences, 119991 Moscow, Russia

*Correspondence: edward@eimb.ru

†These authors contributed equally to this work.

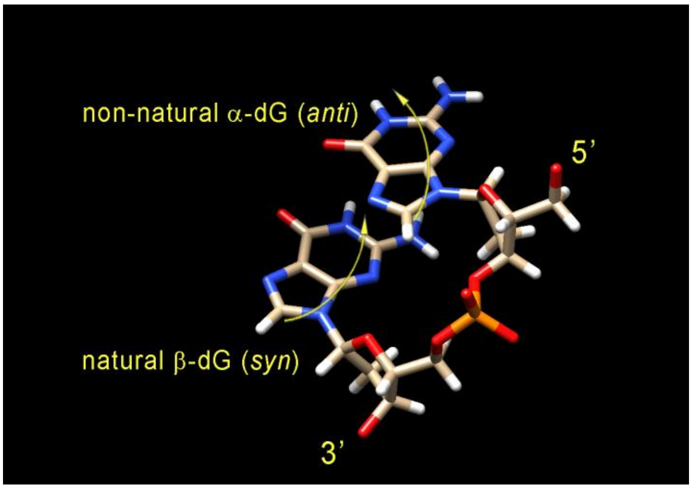

Figure S1. Equivalence of non-natural alpha-dG (*anti*) and natural dG (*syn*) in the context of the G-quadruplex structure. Image generated by UCSF Chimera package.

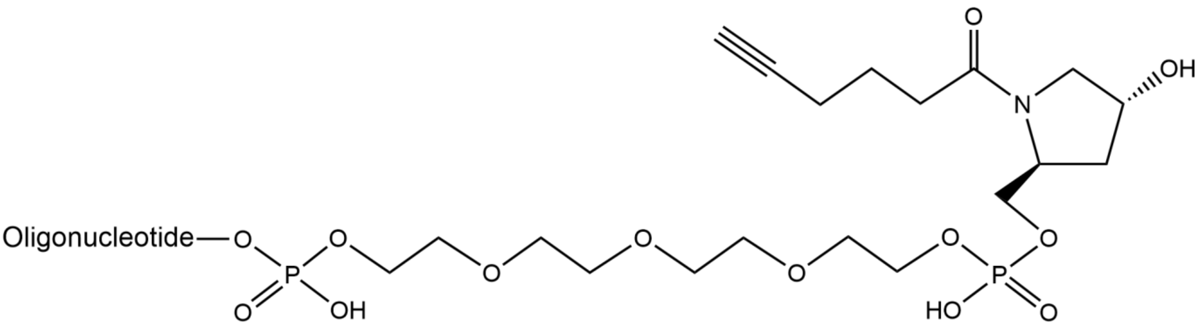

Figure S2. Structure of the clickable 3' cap.

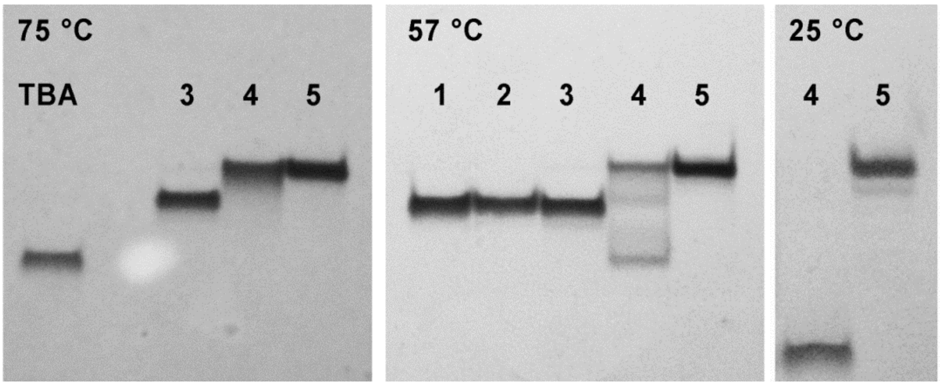

Figure S3. Analysis of modified TBA analogues by electrophoresis in 20% polyacrylamide gel (1 ×TBE buffer) in the presence of 7M urea. The separation time was different in each case. Aptamer 4 retains a stable secondary structure at 25 °C in 7M urea.

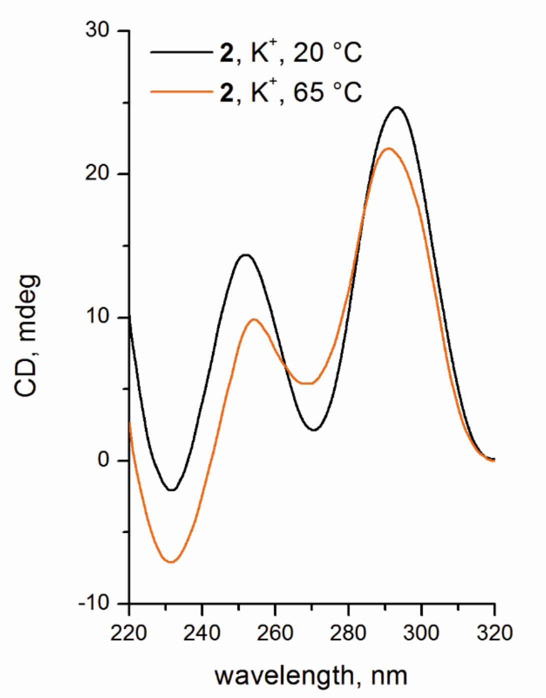

Figure S4. CD spectra of TBA analogue 2 in 10 mM potassium phosphate (pH 7.5) and 90 mM KCl at 20 and 65 °C.

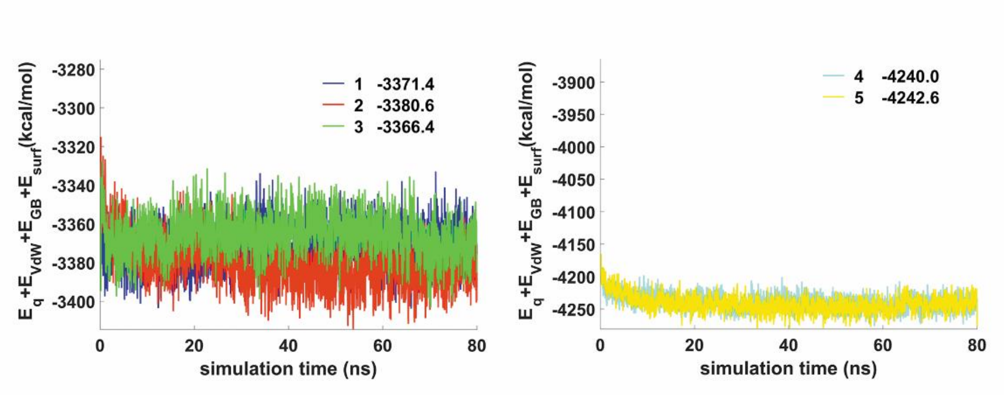

Figure S5. The sum of all contributions to the potential energy for TBA analogues.

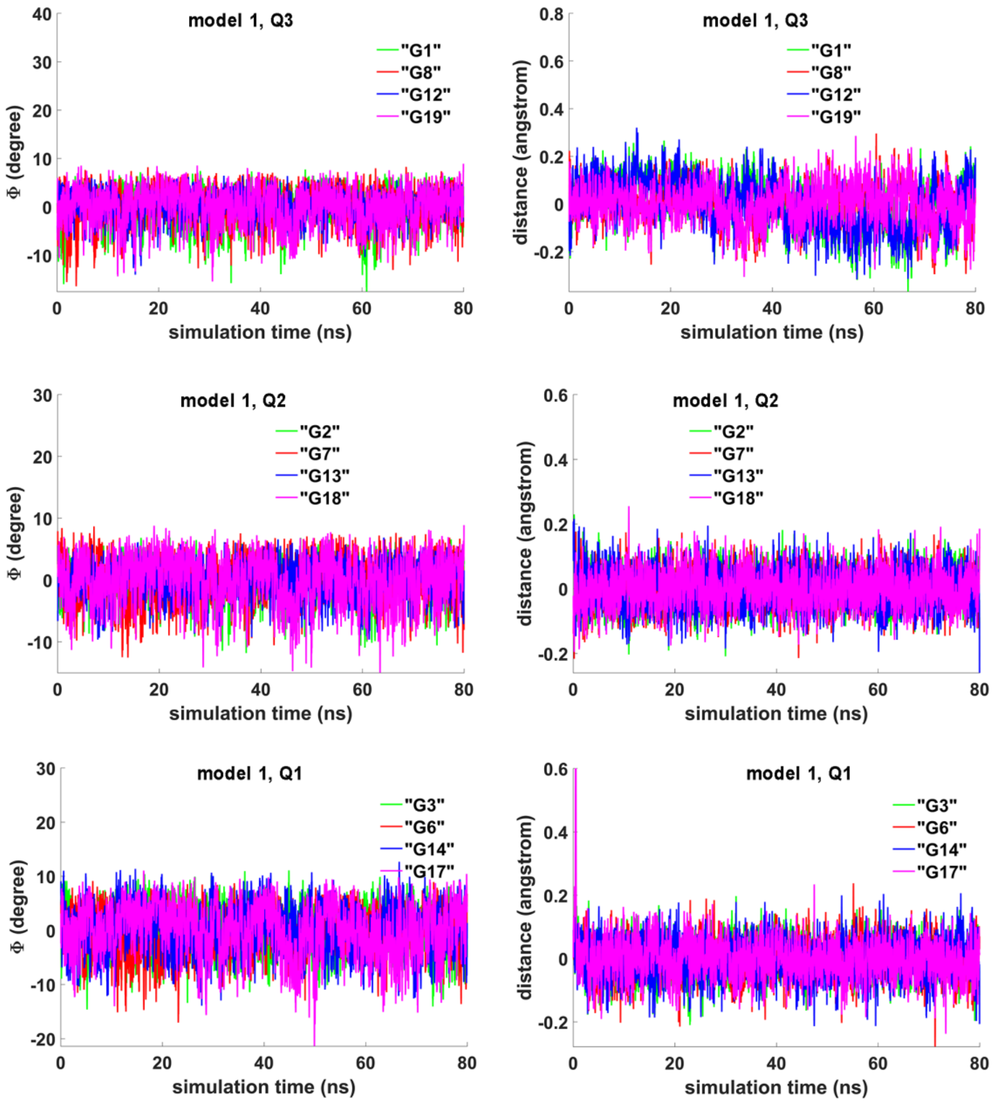

Figure S6. Deviations of geometrical parameters from their average values in tetrads in model 1: the angle between the normal to the tetrad and the normal to a particular guanine (Φ); the distance between the center of mass of the tetrad and the same for a particular guanine.

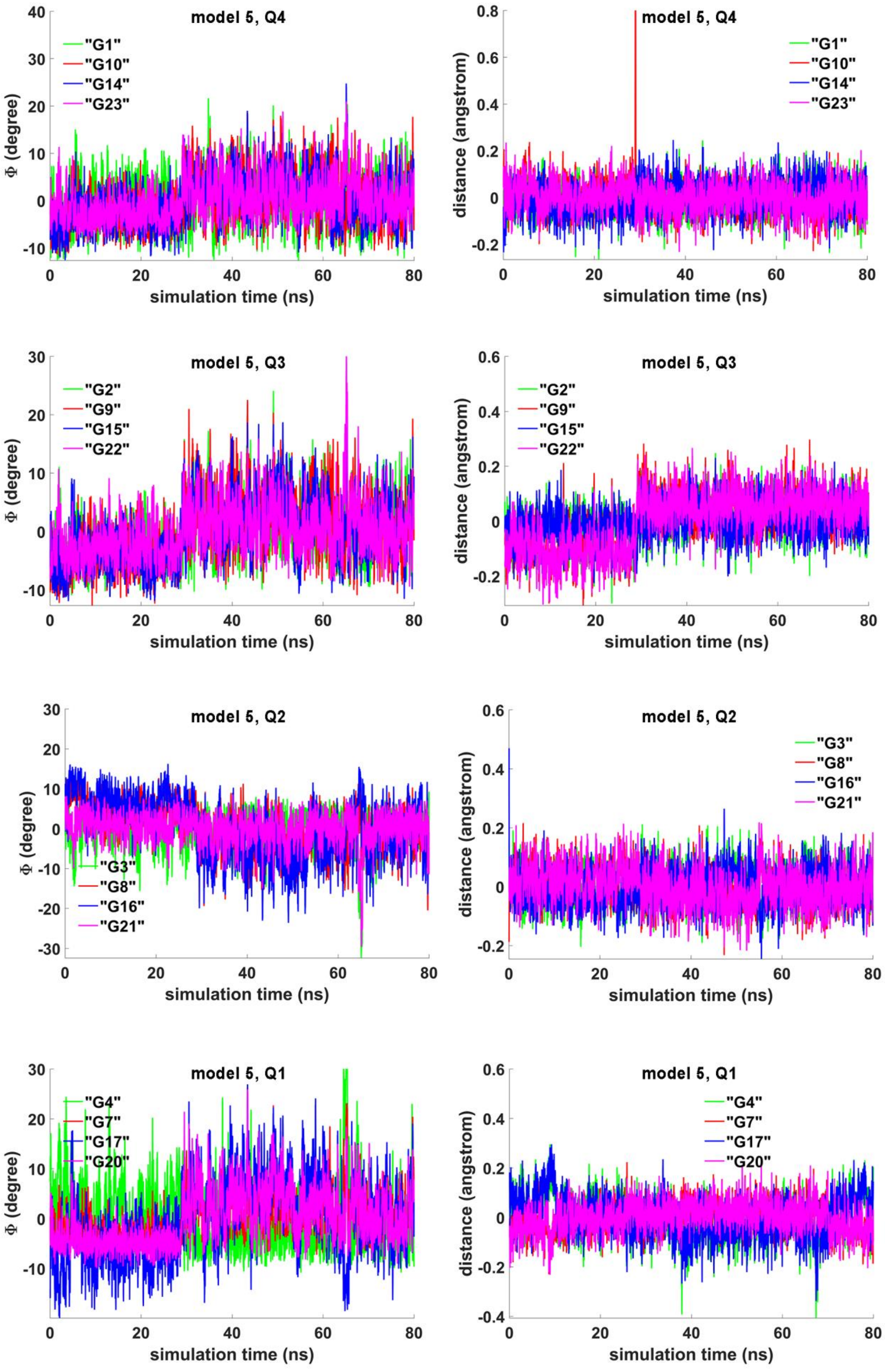

Figure S7. Deviations of geometrical parameters from their average values in tetrads in model 5 (see Figure S6 caption for details).

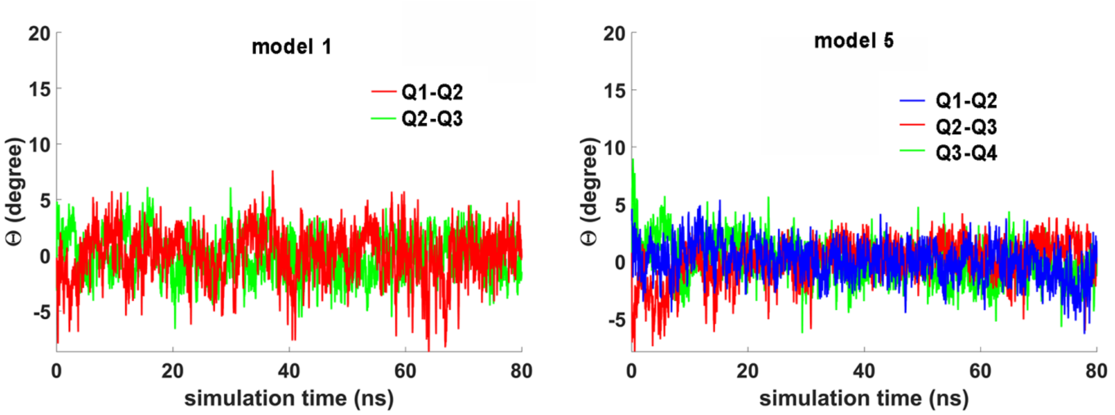

Figure S8. Deviations of the helical twist (θ) from its average value for each pair of tetrads in TBA analogues 1-5.

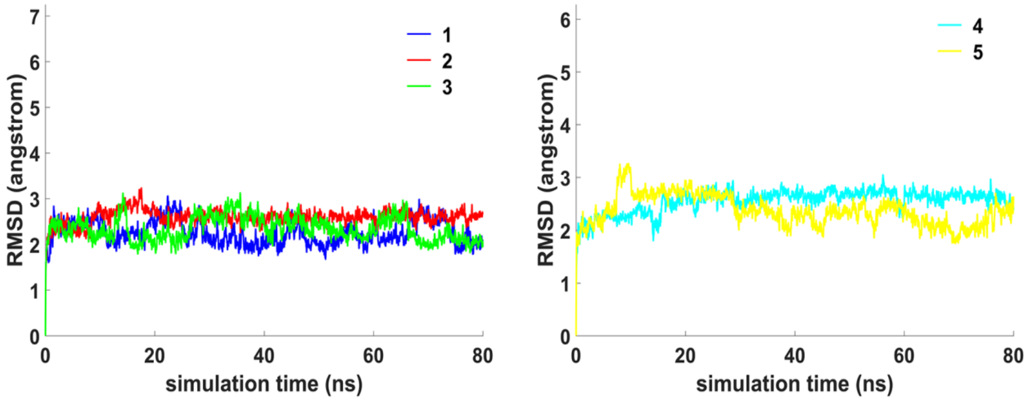

Figure S9. RMSD plots for TBA analogues 1-5.

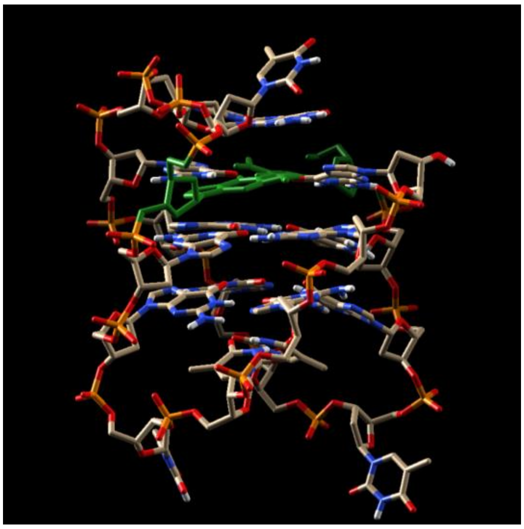

Figure S10. A snapshot of aptamer 2 from the selected angle of view. Modified alpha-dG residues are shown in green. Image generated by UCSF Chimera package.
